# Phytochemical Composition, Anti-Inflammatory Property, and Anti-Atopic Effect of *Chaetomorpha linum* Extract

**DOI:** 10.3390/md22050226

**Published:** 2024-05-17

**Authors:** Luisa Frusciante, Michela Geminiani, Alfonso Trezza, Tommaso Olmastroni, Pierfrancesco Mastroeni, Laura Salvini, Stefania Lamponi, Andrea Bernini, Daniela Grasso, Elena Dreassi, Ottavia Spiga, Annalisa Santucci

**Affiliations:** 1 Dipartimento di Biotecnologie Chimica e Farmacia, Università di Siena, Via Aldo Moro, 53100 Siena, Italy; luisa.frusciante@unisi.it (L.F.); alfonso.trezza2@unisi.it (A.T.); tommaso.olmastroni@student.unisi.it (T.O.); p.mastroeni@student.unisi.it (P.M.); stefania.lamponi@unisi.it (S.L.); andrea.bernini@unisi.it (A.B.); daniela.grasso@student.unisi.it (D.G.); elena.dreassi@unisi.it (E.D.); ottavia.spiga@unisi.it (O.S.); annalisa.santucci@unisi.it (A.S.); 2 SienabioACTIVE, Università di Siena, Via Aldo Moro, 53100 Siena, Italy; 3 Fondazione Toscana Life Sciences, Strada del Petriccio e Belriguardo, 53100 Siena, Italy; l.salvini@toscanalifesciences.org; 4 Advanced Robotics and Enabling Digital TEchnologies & Systems 4.0 (ARTES 4.0), Viale Rinaldo Piaggio, 34, 56025 Pontedera, Italy

**Keywords:** *Chaetomorpha linum*, macroalgae, UPLC-MS/MS, inflammation, RAW 264.7, HaCaT, atopic dermatitis, molecular modeling

## Abstract

Utilizing plant-based resources, particularly their by-products, aligns with sustainability principles and circular bioeconomy, contributing to environmental preservation. The therapeutic potential of plant extracts is garnering increasing interest, and this study aimed to demonstrate promising outcomes from an extract obtained from an underutilized plant waste. *Chaetomorpha linum*, an invasive macroalga found in the Orbetello Lagoon, thrives in eutrophic conditions, forming persistent mats covering approximately 400 hectares since 2005. The biomass of *C. linum* undergoes mechanical harvesting and is treated as waste, requiring significant human efforts and economic resources—A critical concern for municipalities. Despite posing challenges to local ecosystems, the study identified *C. linum* as a natural source of bioactive metabolites. Phytochemical characterization revealed lipids, amino acids, and other compounds with potential anti-inflammatory activity in *C. linum* extract. In vitro assays with LPS-stimulated RAW 264.7 and TNF-α/IFN-γ-stimulated HaCaT cells showed the extract inhibited reactive oxygen species (ROS), nitric oxide (NO), and prostaglandin E2 (PGE2) productions, and reduced inducible nitric oxide synthase (iNOS) and cyclooxygenase-2 (COX-2) expressions via NF-κB nuclear translocation, in RAW 264.7 cells. It also reduced chemokines (TARC/CCL17, RANTES/CCL5, MCP-1/CCL2, and IL-8) and the cytokine IL-1β production in HaCaT cells, suggesting potential as a therapeutic candidate for chronic diseases like atopic dermatitis. Finally, in silico studies indicated palmitic acid as a significant contributor to the observed effect. This research not only uncovered the untapped potential of *C. linum* but also laid the foundation for its integration into the circular bioeconomy, promoting sustainable practices, and innovative applications across various industries.

## 1. Introduction

Marine macroalgae, commonly called seaweeds, and their extracts have become increasingly important in developing nutraceutical products. This is attributed to their substantial content of bioactive compounds, which has captured considerable attention within the pharmaceutical industry as a valuable source of raw materials. Indeed, the high level of biodiversity of marine macroalgae makes them a considerable reservoir for active compounds, given their ability to produce a diverse range of secondary metabolites characterized by a broad spectrum of biological activities [1,2,3,4].

Currently, plant metabolites make up a significant portion of the pharmaceutical industry’s revenue, and using plant materials as a source of bioactive compounds is of great economic importance [5,6]. Natural bioactive compounds derived from marine organisms, particularly invasive algal species, have garnered increased attention in scientific research due to their abundance and wide availability. Seaweeds are the ocean’s living resources. Despite their widespread use in the food and feed industries, they are untapped as nutraceutical and medicinal products despite their significant antioxidative qualities. However, macroalgae are increasingly viewed as a source of secondary metabolites with great potential for developing new drugs. Many previous studies demonstrated the remarkable benefits of macroalgae on human health and protection against chronic diseases due to their content of proteins, lipids, fatty acids, polysaccharides, and antioxidant compounds [2,7,8,9]. Moreover, removing their biomass from invaded environments offers an exceptionally promising potential resource.

This study aimed to investigate the phytochemical composition and biological properties of an extract obtained from the invasive macroalga *Chaetomorpha linum* in LPS-stimulated RAW 264.7 murine macrophages and TNF-α/IFN-γ-stimulated HaCaT human keratinocytes. In silico studies were performed to obtain 3D structures of the entire RAW 264.7 and HaCaT anti-inflammatory target complement, and docking simulations provided findings about potential target/compound interactions. *C. linum* (Müller) Kützing (Basyonim: *Conferva linum* O.F. Müll.) is a macroalgae species in the Chlorophyceae family. It commonly manifests in opportunistic vegetative blooms, thriving in eutrophic environments, especially in ample orthophosphate availability [10]. *C. linum* mats exhibit remarkable resilience and tenacity, presenting significant challenges for their removal. Flourishing since 2005 in the Orbetello Lagoon of Tuscany, Italy, these mats form dense, self-sustaining coverings over approximately 400 hectares. With biomass levels ranging between 2 and 24 kg m^−2^ and the region experiencing mild winters, the mats’ surfaces undergo nearly continuous growth. Their persistence poses challenges for local ecosystems, impacting water quality and the local fishing industry [10,11]. Furthermore, these biomasses are mechanically harvested and treated as waste, requiring significant human efforts and considerable financial resources, which are crucial for local governments. In the Orbetello Lagoon, from 2002 to 2006, an average of 27,098.02 tons (6774 tons per year) of macroalgae were harvested, incurring expenses of about 600,000 Euros per year [12]. However, macroalgae represents a potential resource. 

Various potential uses have been explored for algae from the Orbetello Lagoon over the past decade. Among these, biodiesel production stands out as an intriguing alternative to fossil fuels, offering a sustainable energy supply that reduces greenhouse gas emissions and promotes efficiency [13]. The biomass of macrophytes has been utilized in the past for the production of bio-oil, yielding promising results in terms of extraction efficiency [12]; nonetheless, similar approaches applied to macroalgae from the Orbetello Lagoon have proven less efficient [14]. 

Therefore, this study aimed to contribute to the development of the “circular bioeconomy” mission by exploring possible biotechnological applications for *C. linum* harvested from the Orbetello Lagoon, particularly in the pharmaceutical and cosmeceutical sectors. Inflammatory responses and oxidative stress have recently emerged as significant factors in the pathogenesis and progression of various chronic, non-communicable diseases [15]. Among them, allergic diseases, including allergic rhinitis, food allergy, allergic asthma, atopic dermatitis, and eczema, are systemic conditions whose escalating incidence rates have become a growing concern [16], highlighting the need for comprehensive understanding and effective management strategies [17,18,19]. Common treatments for atopic dermatitis, such as corticosteroids, calcineurin inhibitors, and antihistamines, often result in significant side effects when used over long periods [20,21,22,23]. This emphasizes the potential of natural plant-derived remedies, which offer effective treatment with minimal adverse effects. Such botanical alternatives present a safer option for managing skin inflammation, with the potential to reduce or eliminate the occurrence of side effects [24]. In recent years, there has been a steady increase in the prevalence of allergic diseases [25,26,27]. Evidence suggests that cutaneous inflammatory conditions typically progress to more systemic inflammatory diseases such as allergic rhinitis and asthma, a phenomenon referred to as the “atopic march” [28,29,30]. Furthermore, the growing field of “cosmeceuticals”, a recently coined term to describe products that blend cosmetic and pharmaceutical properties, refers to products capable of enhancing or altering skin functions and appearance, thereby providing skin benefits [31]. This expanding sector is renowned for its innovative approach, constantly searching for active molecules with improved properties to counteract undesired effects, primarily by integrating natural resources into cosmetic formulations. Natural resources present numerous advantages, including environmental friendliness, reduced toxicity, non-carcinogenic properties, accessibility, minimal side effects, and economic benefits [32,33]. With a specific focus on its anti-inflammatory properties, the objective was to repurpose and add value to *C. linum’s* strategic and valuable biomass.

## 2. Results

### 2.1. Chemical Composition of C. linum Extract

The extraction process plays a key role in the recovery of biomolecules from natural matrices, as it affects their quality and quantity. There are several methodologies applicable for the extraction of bioactive compounds, and their choice is based on the chemical and physical characteristics of the molecules of interest as well as the starting raw material. Heat-reflux extraction of 10 g of oven-dried pulverized *C. linum* algae collected from the Orbetello lagoon with an ethanol–water (70:30 *v*/*v*) mixture produced 1.247 g of freeze-dried *C. linum* ethanolic (CLE) dry extract, resulting in a percentage yield of 12.47% (*w*/*w*). In order to assess the effectiveness of ethanol–water extraction, the crude extracts were subjected to NMR testing, where the molecular classes and relative fractions were closely examined. Proton NMR spectrum revealed the presence of both saturated and unsaturated fatty acids through a set of broad peaks that had a higher intensity (Figure S1) when compared to the peaks of polar species. Although peaks indicating amino acids, sugar, and aromatics were also detected, their presence was much lesser. These results suggested that the CLE dry extract was significantly enriched in fatty acid contents as compared to polar metabolites. Based on this evidence, further characterization of the mixture by proper separation methods was initiated.

#### 2.1.1. UPLC-MS/MS Analysis

CLE extract was profiled by UPLC-MS/MS. A total of 19 metabolites were identified using Compound Discoverer 3.3 software integrated using the ChemSpider database and the mzCloud for data processing and compared with the literature data [34,35]. Overall, most of the metabolites detected in CLE were lipids, with compounds identified as free fatty acids (MUFA 18:1ω-9 Oleic acid, PUFA 18:4ω-3 stearidonic acid) and fatty acids derivatives. Matched metabolites were listed in Table 1, along with their retention time, molecular formulae, observed and theoretical *m*/*z*, and error (ppm). For all matched compounds, the error was lower than 5 ppm. Among them, one was a palmitic acid monoacylglycerol (MAG)-derivative (Palmitin), three were identified as hydroxy-derivatives of saturated fatty acids (Hydroxymyristic acid, Hydroxylauric acid, and Dihydroxypalmitic acid), two PUFA 18:3n-3 Alpha-linoleic acid-derived oxylipins (9(10)-EpODE, Hydroxylinoleic acid), a fatty acid amide (Lauramide), and a fatty aldehyde (8-Pentadecenal). The other matched metabolites were identified as amino acids (Valine, Norleucine, and Thymine), terpenes (Carnosic acid, Rosmanol, and Methyl dehydroabietate), and flavonoids (Kaempferol, Apigenin, and 3′-O-Methylequol). 

#### 2.1.2. GC-FID Analysis

**Figure 1:**
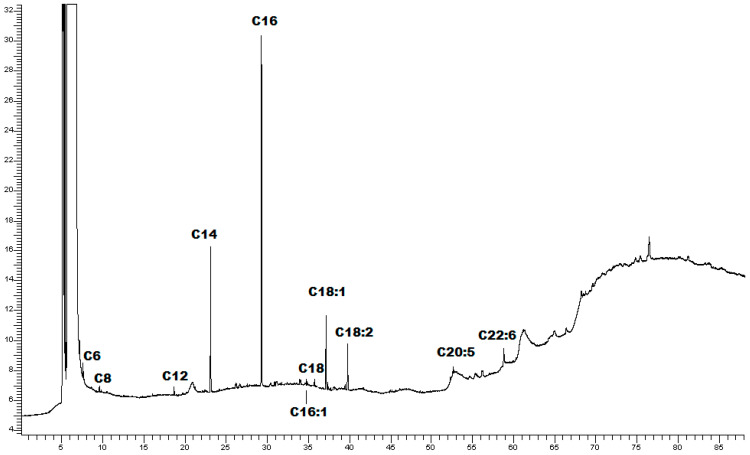
Chromatographic profile of CLE.

CLE fatty acid pattern was further evaluated by GC-FID. The chromatographic profile and fatty acid methyl esters identified in CLE were reported in Figure 1 and Table 2, respectively. 

The analysis revealed that the predominant fatty acid in CLE was palmitic acid (16:0) (52.18%), followed by myristic acid (14:0) (20.46%), and oleic acid (18:1ω9) (11.70%).

### 2.2. The Effect of CLE on Cell Viability of RAW 264.7 and HaCaT Cells

**Figure 2:**
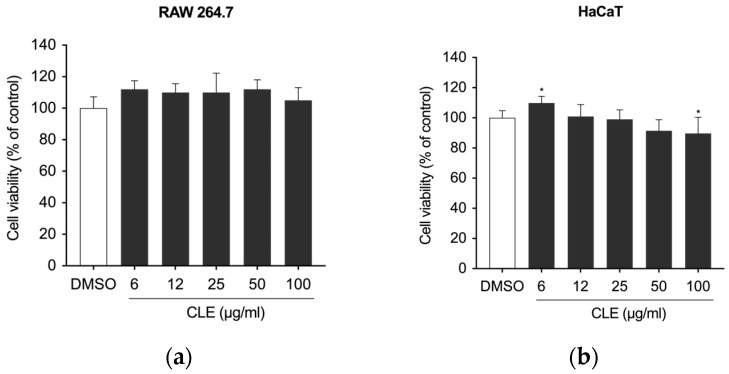
The effects of CLE on the viability of (**a**) RAW 264.7 and (**b**) HaCaT cells. After culturing the cells with CLE (0, 6, 12, 25, 50, and 100 μg/mL), cell viability was measured with the MTT and CCK-8 assays, respectively, after 24 h. All data showed mean ± SD values of three independent experiments. Statistically significant differences were denoted by * *p* ≤ 0.0207 (vs. DMSO). *p*-values were calculated using one-way ANOVA with Dunnett’s post hoc test.

First, to exclude any cytotoxic effect of CLE, the viability of RAW 264.7 and HaCaT cells was measured with MTT and CCK-8 assay, respectively. The findings indicated that CLE was not cytotoxic at concentrations up to 100 µg/mL; a minor reduction in viability was observed at a concentration of 100 μg/mL after 24 h of treatment (Figure 2).

### 2.3. The Effects of CLE on Inflammatory Mediators in LPS-Stimulated RAW 264.7 Cells

**Figure 3:**
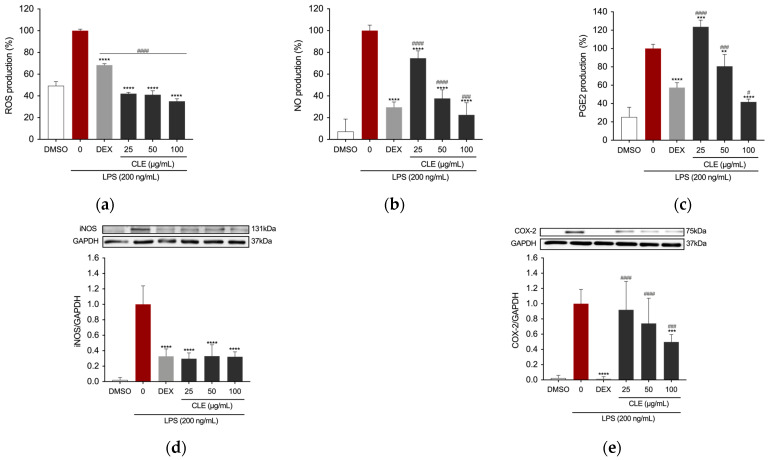
The effects of CLE on LPS-induced ROS, NO, and PGE2 production, and iNOS and COX-2 protein expression levels in RAW264.7 cells: (**a**) Intracellular ROS level was quantified after pre-treatment with different concentrations of CLE followed by LPS stimulation (200 ng/mL) for 5 h. Data were presented as bar graphs for ROS level measured from relative fluorescence intensity normalized to cell count with Crystal Violet assay. Culture supernatants of RAW 264.7 cells pre-treated with DEX or CLE for 4 h and then stimulated with 200 ng/mL LPS for 24 h were analyzed for (**b**) NO and (**c**) PGE2 production. iNOS (**d**) and COX-2 (**e**) expression levels were determined by Western blotting. All data showed mean ± SD values of three independent experiments. Statistically significant differences were denoted by ** *p* = 0.0039, *** *p* = 0.0005, and **** *p* < 0.0001 (vs. LPS). ^#^ *p* = 0.0231, ^###^ *p* ≤ 0.0009, and ^####^ *p* < 0.0001 (vs. DEX as positive control). *p*-values were calculated using one-way ANOVA with Tukey’s post hoc test.

Reactive oxygen species (ROS) serve as signaling molecules crucial in the development of inflammatory disorders [36]. Therefore, we investigated the impact of pretreating RAW 264.7 cells stimulated with LPS with CLE on intracellular ROS production. Dexamethasone (DEX) at a concentration of 5 µg/mL was chosen as a reference for assessing anti-inflammatory efficacy. The cells were pre-treated with DEX and with varying concentrations of CLE (25, 50, and 100 µg/mL) before being stimulated with LPS. Treatment with DEX resulted in a statistically significant reduction in ROS production level in stimulated RAW 264.7 cells (Figure 3a), a decrease that was also evident following treatment with all tested concentrations of CLE. Notably, CLE demonstrated a significantly superior effect compared to 5 µg/mL DEX at all concentrations. 

A key response of RAW 264.7 cells to inflammatory stimuli was the secretion of pro-inflammatory cytokines and mediators, with nitric oxide (NO) being particularly notable. To assess CLE’s anti-inflammatory properties, we measured NO levels in the culture supernatants of both untreated and CLE-treated LPS-stimulated RAW 264.7 cells with Griess assay. Positive control was achieved by treating the cells as described previously. Cells treated with LPS exhibited elevated levels of NO. Conversely, in the presence of CLE, there was a concentration-dependent reduction in NO production (Figure 3b). Specifically, CLE at concentrations of 25 and 50 μg/mL significantly reduced NO levels in LPS-stimulated RAW cells. Furthermore, treatment with 100 µg/mL CLE resulted in a decrease in NO level significantly greater than the positive control. Interestingly, CLE at concentrations of 50 and 100 µg/mL also inhibited PGE2 level in the supernatant of RAW 264.7 cells, with the highest concentration tested exhibiting an effect superior to the positive control (Figure 3c).

To further investigate the anti-inflammatory mechanism of CLE, the protein expression of iNOS and COX-2, precursor enzymes of NO and PGE2, respectively, was assessed using Western blotting. As reported in Figure 3d,e, the expression of iNOS and COX-2 was upregulated in the LPS-treated group. However, this increase was inhibited by CLE.

### 2.4. The Effect of CLE on NF-κB Activation in LPS-Stimulated RAW 264.7 Cells

**Figure 4:**
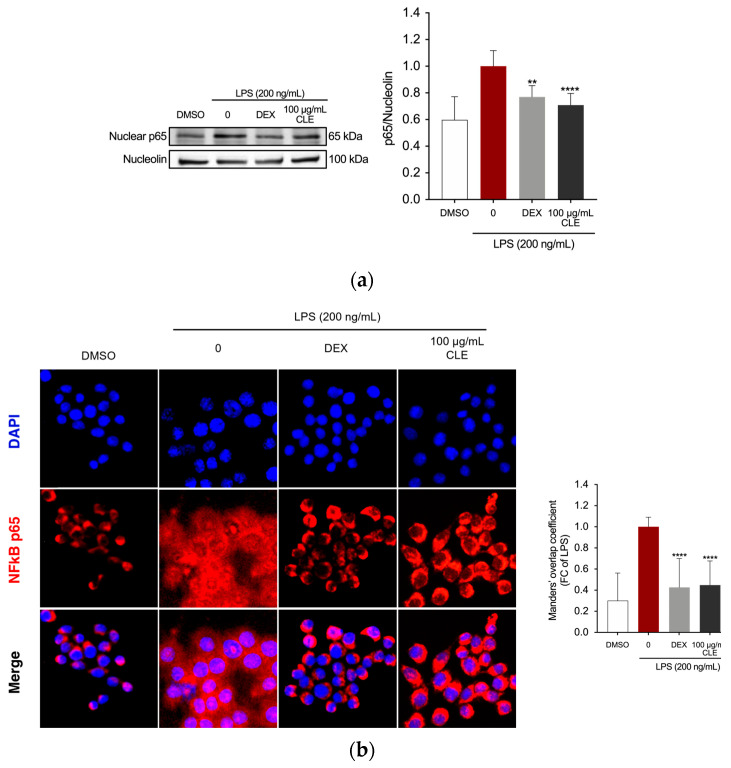
The effects of CLE on suppressing the upstream signaling for NF-κB activation in LPS-induced RAW264.7 cells. Cells were pre-treated with DEX or CLE for 4 h and then incubated with LPS (200 ng/mL) for 1 h. (**a**) NF-κB in LPS-stimulated RAW264.7 cells using Western blotting. Quantification of relative band intensities from three independent experimental results determined by densitometry. Data were presented as mean ± SD of three independent experiments. ** *p* = 0.016, **** *p* < 0.0001 (vs. LPS). (**b**) Localization of NF-κB visualized by a fluorescent microscope after staining for NF-κB (red). The nuclei of cells were stained with DAPI (blue). The bar graph shows the quantification (using Manders’ coefficient) of NF-κB p65 co-localization with DAPI. **** *p* < 0.0001 (vs. LPS). Micrographs were captured with 40× magnification.

The study examined whether CLE could hamper the activation of the NF-κB pathway, as the regulation of inflammatory mediators in LPS-stimulated macrophages involves NF-κB activated transcriptional processes [37]. Western blotting for NF-κB was conducted to verify whether CLE can suppress the nuclear translocation of NF-κB. Exposure to LPS alone increased the amount of NF-κB in the nucleus (Figure 4a). However, CLE at 100 μg/mL significantly inhibited increased levels of nuclear NF-κB p65 compared to the LPS group, with an effect comparable to that of DEX positive control (*p* = 0.801). The analysis of NF-κB localization by immunofluorescence staining and colocalization analysis confirmed that in untreated RAW 264.7 cells, the p65 protein was localized outside the nucleus. LPS stimulation induced the translocation of p65 from outside to inside the nucleus. However, DEX and CLE retained NF-κB in the cytoplasm of cells (Figure 4b).

### 2.5. CLE Reduced Inflammation in TNF-α/IFN-γ Insulted HaCaT Cells

**Figure 5:**
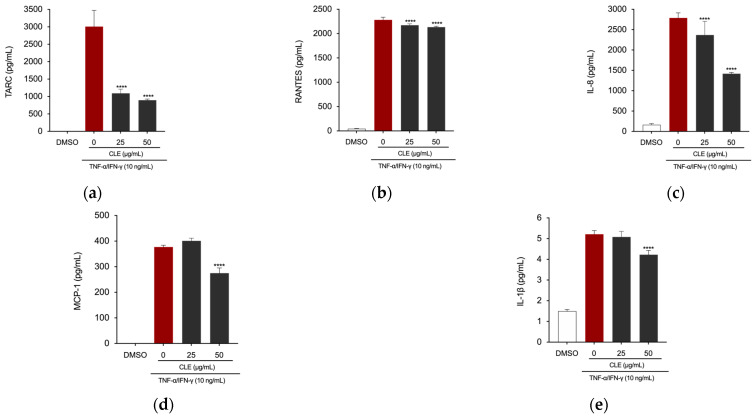
The effects of CLE on TNF-α/INF-γ-induced production of pro-inflammatory chemokines (upper) and cytokines (lower) in HaCaT cells. After pre-treating the cells with DEX or CLE for 4 h, cells were stimulated with 10 ng/mL TNF-α/INF-γ for 24 h. Levels of (**a**) TARC/CCL17, (**b**) RANTES/CCL5, (**c**) IL-8, (**d**) MCP-1/CCL2, and (**e**) IL-1β were measured using ELISA kits. Data showed mean ± SD values of three independent experiments. **** *p* < 0.0001 (vs. 10 ng/mL TNF-α/INF-γ). *p*-values were calculated using one-way ANOVA with Dunnett’s post hoc test.

To investigate the therapeutic potential of CLE against skin inflammatory disorders, we assessed its protective effect on inflammatory responses in HaCaT human keratinocytes. Considering the results obtained from the CCK-8 assay, which indicated a slight decrease in cell viability of HaCaT cells following treatment with CLE at a concentration of 100 µg/mL, we proceeded with our investigations only using concentrations of 25 and 50 µg/mL. The cells were pretreated with concentrations of CLE for 4 h, followed by treatment with TNF-α/IFN-γ at 10 ng/mL for 24 h. Treatment with CLE at concentrations of 25 and 50 µg/mL significantly decreased the production of TARC/CCL17, RANTES/CCL5, and IL-8 in the supernatant of TNF-α/IFN-γ-induced HaCaT cells (Figure 5a–c). Additionally, the concentration of 50 µg/mL was capable of reducing the levels of the chemokine MCP-1/CCL2 and the cytokine IL-1β (Figure 5d,e).

### 2.6. Mutagenicity Assay: Ames Test

**Figure 6:**
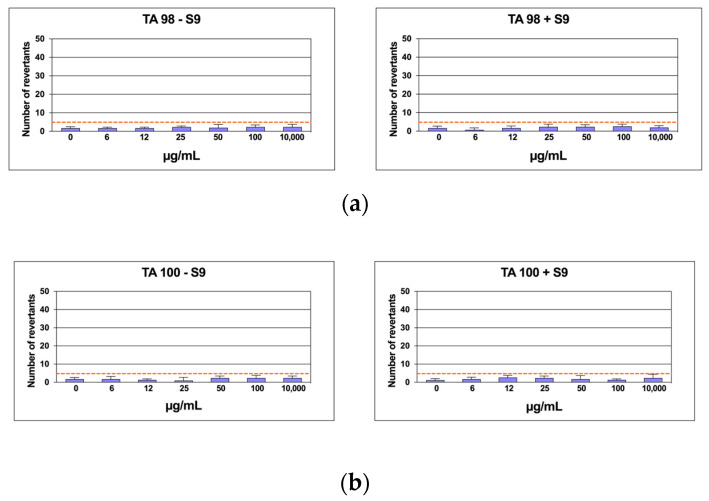
The number of revertants in TA98 (**a**) and TA100 (**b**) *S. typhimurium* strain exposed to different concentrations of CLE with S9 fraction and without S9 fraction. The results were reported as the mean of revertants ± SD; *n* = 6; *p* ≤ 0.01.

Six distinct CLE concentrations were evaluated by the Ames test on TA98 and TA100 bacteria, both with and without S9 metabolic activation, in the Salmonella mutagenicity assay. The findings regarding the mutagenic impact of the specimens, as reported in Figure 6a,b, showed that CLE was non-genotoxic to TA98 and TA100 at all concentrations examined, both in the presence and absence of S9 fraction. Indeed, the number of revertants was smaller and significantly different from the positive control at the maximum concentration (1000 µg/mL) (*p* ≤ 0.01). In every instance, the background level and positive control levels fell within the typical range observed in our lab.

### 2.7. In Silico Results

#### Target/Compound Virtual Screening

To identify potential targets involved in the interaction with CLE compounds, a ligand-based virtual screening was performed against the entire RAW264.7 and HaCaT cells anti-inflammatory target complement identified from the “target section” in the DrugBank database. Our study identified 53 targets indirectly or directly involved with the cell’s anti-inflammatory condition for both cell lines. Each primary structure was downloaded from the UniProt database. To verify the availability of target 3D structures, a multiple sequence alignment (MSA) was performed using BLASTp against the “Protein Data Bank” database. Based on the MSA results, we obtained and downloaded a total of 35 protein 3D structures (Table S1). 

**Figure 7:**
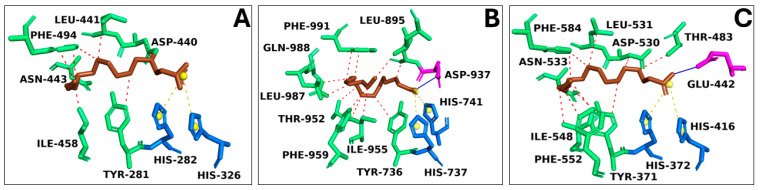
The overview of target/palmitic acid complexes. Three-dimensional structures of (**A**) PDE4C2 (PDB code: 2QYM), (**B**) PDE3B (PDB code: 1SO2), and (**C**) PDE4A10 (PDB code: 2QYK), in complex with palmitic acid represented in brown sticks. The binding residues involved in hydrophobic interactions, hydrogen bonds, and salt bridges were represented as green, pink, and blue sticks, respectively. Hydrophobic interactions were reported as red dotted lines, hydrogen bonds as a continuous blue line, and salt bridges as yellow dotted lines, with the charge center represented as a yellow sphere.

Each structure was optimized through molecular modeling to resolve potential structural gaps and steric clashes; subsequently, virtual screening was performed among all targets and compounds extracted from our natural source. To standardize our analyses and enhance the reliability of our in silico results, we adopted two different strategies to select the best three complexes: (i) binding free energy (docking score) and (ii) evolution approach considering the interaction network consensus binding residues, as suggested in previous work [38]. As a result of the virtual screening, we selected the first three complexes with the highest binding free energy: the catalytic domain of human phosphodiesterase 3b (PDB code: 1SO2), the crystal structure of PDE4A10 (PDB code: 2QYK), and the crystal structure of PDE4C2 (PDB code: 2QYM). In detail, 1SO2/PA, 2QYK/PA, and 2QYM/PA complexes showed binding energies of −6.9 kcal/mol, −6.7 kcal/mol, and −6.5 kcal/mol, respectively. Interaction network analyses revealed that palmitic acid formed a wide polar and hydrophobic interaction network within the target binding pocket (Figure 7).

## 3. Discussion

The importance of developing new anti-inflammatory agents from natural sources is widely acknowledged today and represents a critical imperative to pursue. Seaweeds, in this regard, have been suggested as promising anti-inflammatory candidates due to their composition of lipids, phenolic compounds, carotenoids, phytosterols, alkaloids, and polysaccharides. In this study, we evaluated the anti-inflammatory activity and phytochemical composition of a hydroalcoholic extract obtained from the green macroalga *C. linum*, an invasive species in the Orbetello Lagoon (Tuscany, Italy), to determine the potential reuse of this “waste” that would otherwise require costly biomass treatment and management.

Plants can produce a variety of secondary (specialized) metabolites with significant biological activity due to their need to adapt to environmental changes. This is especially true for algae, which must ensure survival in one of the harshest and everchanging environments. As a result, even within the same species or depending on meteorological conditions and harvesting period, the production of metabolites varies significantly [3]. Because of this characteristic, analyzing the chemical composition of algae is particularly challenging. Yet, it makes them ideal contenders for screening candidate compounds with bioactive potential. While data concerning the anti-inflammatory activity of *C. linum* species remains limited, and comprehensive investigations into the phytochemical composition of *C. linum* from the Orbetello Lagoon are lacking; previous studies examined samples from various regions of the Mediterranean Sea for their lipid content. These studies reported different lipidic profiles for *C. linum* from different origins [7,8,35,39,40,41], thereby positioning it as an attractive candidate for further exploration of its potential anti-inflammatory properties. 

Recent studies on *Chlorophyta* of genus Ulva, widely utilized as a model for investigating complex metabolic networks due to their rapid growth rate and ability to thrive under diverse environmental conditions, have shed light on changes in metabolic pathways involved in lipid biosynthesis. These pathways are utilized by algae to adapt to conditions of nutritional stress, particularly from nitrates and phosphates [42,43].

The phytochemical composition of CLE was evaluated using UPLC-MS/MS. The most prevalent class of secondary metabolites found in CLE was lipids. GC-FID analysis further indicated that the predominant fatty acid in CLE was palmitic acid (16:0) (52.18%), followed by myristic acid (14:0) (20.46%), and oleic acid (18:1ω9) (11.70%). In a study by Biandolino et al., *C. linum* collected from Mar Piccolo of Taranto was reported to contain both saturated and unsaturated fatty acids, with unsaturated fatty acids representing the predominant part. Among them, C18 PUFAs were the dominant unsaturated fatty acids. Palmitic acid (16:0) was the dominant saturated species, with a high proportion of myristic acid (14:0), whereas MUFAs were primarily represented by 18:1ω9 and 18:1ω7 [41]. Another study on *C. linum* from the Sea of Japan reported a percentage of MUFAs primarily represented by 18:1ω9 and 18:1ω7, but the highest proportion of saturated fatty acids was in that case of myristic acid (14:0) [44]. Extracts from *C. linum* growing wild in Corsican Pond, on the other hand, report saturated fatty acids as the main compounds from a pentane *C. linum* extract, whereas the components of the sterol’s family were the major compounds from an ethyl acetate extract [35]. Differences may be attributed to the extraction method used for the analysis, but ecological conditions, the life cycle of the algae, and seasonal variations are also of extreme importance. The nutritional composition of several *Clorophyta* species from fishpond aquaculture systems, including *C. linum*, was also studied, along with their anti-inflammatory activities and the effects that their bioaccessibility could have on this property. It was found that in *C. linum*, the total PUFA content was low, and a high level of saturated fatty acids balanced this low PUFA content. However, of all the tested species, *C. linum* was the one with the highest ω3/ω6 ratio [7]. Among the unsaturated fatty acids, the most abundant ω3 PUFA was α-linolenic acid (18:3ω3) along with MUFA 18:1, while myristic (14:0) and palmitic (16:0) were the dominant saturated species. Moreover, in the same study, *C. linum* demonstrated the highest inhibitory activity on COX-2. The anti-inflammatory activity of *C. linum* and its bioaccessible fraction was measured as a percentage of COX-2 inhibition [7]. At 100 μg/mL, the extract displayed inhibitory properties in accordance with other works using the same COX-2 inhibition methodology [45]. It should be noted that following in vitro digestion studies, they found low bioaccessibility of the compounds under investigation for their anti-inflammatory activity, recommending that the seaweed be used as an extract and not as food so that the destruction of cell walls would make bioactive compounds more bioaccessible [7]. Seaweed-derived lipids have been extensively studied for their anti-inflammatory, antioxidant, antimicrobic, antitumoral, and cardioprotective properties [46]. The anti-inflammatory effect of lipid extracts from both microalgae and macroalgae is well-recognized, with *Ochrophyta* and *Rhodophyta* being the most investigated phyla among the macroalgae [47,48,49,50]. Nevertheless, the *Chlorophyta* phylum has, to date, received little attention on this side [51,52]. Investigations were primarily conducted in vitro using murine macrophage RAW 264.7 and human THP-1 monocytic cell lines, with COX-2 activity and NO level being the most often assessed parameters. Very few examples of in vivo tests are reported. Unsaturated fatty acids, and especially ω-3 PUFAs, are thought to be primarily responsible for the anti-inflammatory activity of seaweeds [48,53]. Notoriously, macroalgae, despite having a relatively modest (0.5% to 8.0% of DW) lipid content, are the main primary producers of both ω-3 and ω-6 PUFAs [2]. Lipid crude extracts, lipid fractions, or isolated complex lipids are typically produced using a methanol/chloroform (2:1) mixture [2], but other organic solvents have demonstrated good efficiency in extracting lipids from algal samples. For this study, we evaluated the practicability of analyzing the composition of *C. linum* using less aggressive and easier-to-handle solvents, opting for a mixture of ethanol and water (70:30 *v*/*v*), since the literature data report lipid-enriched extracts with similar solvents [39,54,55]. Despite having higher extraction efficiency, other organic solvents such as hexane, chloroform, and methanol have the drawback of being very toxic. The European Parliament and Council Directive 2009/32/EC of 23 April 2009 for the approximation of the laws of the Member States concerning the extraction solvents used in the preparation of food products and their “ingredients” establishes that ethanol and acetone can be used without concentration limits. Acetone and ethanol are part of the solvents defined as GRAS (Generally Recognized as Safe) since toxicological studies show no long-term adverse effects on human health. Acetone is considered a GRAS by the FDA on par with ethanol, but only ethanol can be used without any concentration limits [56,57]. 

Potential anti-inflammatory properties of *C. linum* ethanolic extract were assessed in LPS-stimulated RAW 264.7 macrophage cells and TNF-α/IFN-γ-stimulated HaCaT keratinocyte cells. According to the findings of the current investigation, CLE had no cytotoxic effects and did not dramatically influence the viability of macrophages or keratinocytes at any of the tested concentrations. Additionally, it was not found to be mutagenic. During the inflammatory process, the major functions of macrophages are antigen presentation, phagocytosis, and immunomodulation through the production of various cytokines and growth factors [21]. It is well known that iNOS and COX-2 play an important role in inflammation. Additionally, ROS acts as a crucial signaling molecules in the development of inflammatory disorders [36]. NO and PGE2, downstream signaling factors of iNOS and COX-2, and several other pro-inflammatory cytokines and chemokines, are involved in the regulation of immune and inflammatory responses, causing symptoms such as pain, fever, and edema [58]. NO, which is regulated by iNOS, is a potent reactive factor in inflammatory responses found in stimulated macrophages and in the sites of inflammation [59]. COX-2 catalyzes the conversion of arachidonic acid to prostaglandins, including PGE2, and COX-2 activity can contribute to inflammatory pain. All these molecular players, in concert, may induce the recruitment of further inflammatory cells, resulting in triggering acute generalized inflammatory responses characteristic of septic shock and multi-organ failure [60]. For this reason, therapeutic interventions that target macrophages and their products could open new avenues for anti-inflammatory treatments. Therefore, we evaluated the anti-inflammatory effects of CLE by measuring the levels of these inflammation-related factors in RAW 264.7 cells. Pre-treatment with CLE significantly reduced protein expression of LPS-induced increased levels of iNOS and COX-2 and the release of ROS, NO, and PGE2 in RAW 264.7 cells in a dose-dependent manner. Targeted inhibition of COX-2 is a promising approach to inhibit inflammation, with phytonutrients and phytochemicals holding the potential to act in this regulation.

NF-κB plays an important role in the control of gene-encoding pro-inflammatory cytokines, as well as inducible enzymes, including iNOS and COX-2 [37]. It comprises five members, c-Rel, p65/RelA, RelB, p50/NF-κB1, and p52/NF-κB2, which form various homo- or heterodimers to control target gene transcription [37]. While inactive, NF-κB remains in the cytoplasm bound to an inhibitor of κB (IκB) proteins. Cellular activation triggers IKK activation, leading to IκB phosphorylation and its dissociation from p65 NF-κB, thereby activating NF-κB. The activated NF-κB complex moves to the nucleus, where it binds to NF-κB-binding sites in gene promoters, regulating their expression [61]. Increased activation of the NFκB signaling pathway triggers the production of downstream inflammation-related factors. Since we found that CLE modulates NF-κB downstream pro-inflammatory markers, we further elucidated if CLE could interfere with the activation of NF-κB signaling by examining its effect on inhibiting LPS-induced translocation of NF-κB into the nucleus. Our results showed that LPS treatment caused a significant decrease in NF-κB in the nucleus, but this effect could be reversed by pre-treatment with CLE, indicating that signal transduction pathways mediated by NFκB may be effectively blocked by CLE in activated macrophages. 

Keratinocytes, a prominent group of epidermal cells crucial in the development of inflammatory skin lesions [62], and macrophages engage in crosstalk to serve as the primary defense against infections and regulate the skin’s homeostatic and inflammatory responses. Beyond their physical barrier function, keratinocytes actively contribute to the pathophysiology of various skin diseases by working with macrophages to regulate inflammatory responses [63,64]. Keratinocytes release mediators that stimulate blood-borne monocytes to differentiate into M1 macrophages, which release pro-inflammatory cytokines that activate keratinocytes. The role of macrophages in the inflammatory process and the use of RAW 264.7 cells as a suitable in vitro model to study inflammation have been well established. Our extract effectively reduced LPS-induced ROS generation in RAW 264.7 macrophages. Oxidative stress is a central player in inflammatory conditions, as evidenced by recent research linking it to the pathogenesis of atopic dermatitis (AD) in humans [65]. Stressed keratinocytes release various chemokines like IL-8, which recruit neutrophils. ROS activation triggers NF-κB, modulating genes such as TNF-α, interleukins, and iNOS [66]. Skin inflammation induced by oxidative stress involves the release of inflammatory mediators like COX-2 and TNF-α, regulated by signaling pathways including NF-κB, MAPK, and JAK/STAT [67]. Our finding that CLE inhibited ROS generation, as well as the production of NO and PGE2 in the supernatant of stimulated RAW 264.7 cells and their progenitor enzymes, presented promising avenues for treating inflammatory skin conditions, including AD.

TNF-α and IFN-γ are pro-inflammatory mediators secreted by macrophages that can stimulate keratinocytes, activating various cell signaling mechanisms and increasing the expression of pro-inflammatory mediators in keratinocytes [68,69]. HaCaT keratinocyte cells stimulated with TNF-α/IFN-γ are a commonly used in vitro model for studying inflammatory skin diseases [70]. TNF-α and IFN-γ can work synergistically to induce the expression of inflammatory cytokines (IL-6 and IL-1β) and chemokines (TARC/CCL17, MCP-1/CCL2, IL-8, and RANTES/CCL5) in HaCaT keratinocytes [71]. Cytokines are well-known for mediating inflammatory cell migration, keratinocyte proliferation, and further cytokine production by keratinocytes. Prominent players in these processes include IL-1β and IL-6, while key inflammatory chemokines such as RANTES/CCL5 and TARC/CCL17 orchestrate the recruitment of leukocytes to inflammation sites. Moreover, MCP-1/CCL2 and IL-8 are closely linked to the onset and severity of chronic skin inflammation, with TARC/CCL17 levels showing a positive correlation with atopic dermatitis severity in affected individuals [71]. RANTES/CCL5 predominantly acts as a chemotactic agent, activating T-cells [61]. The NF-κB family, consisting of transcription factors activated by various stimuli, including LPS, TNF-α, and IFN-γ, plays a critical role in the expression of multiple proinflammatory genes [61]. These genes regulate the production of proinflammatory cytokines like IL-6 and IL-1β, as well as chemokines such as TARC/CCL17, RANTES/CCL5, and MPC-1/CCL2 in HaCaT cells [62]. 

In addition to reducing the production of inflammatory mediators in LPS-stimulated RAW 264.7 macrophage cells, potentially by inhibiting NF-κB nuclear translocation, CLE also dose-dependently decreased the levels of chemokines (TARC/CCL17, RANTES/CCL5, MCP-1/CCL2, and IL-8) and the cytokine IL-1β in TNF-α/IFN-γ-induced HaCaT cells. Considering these findings, the discovery that the ethanolic extract from *C. linum* biomass can significantly suppress key inflammatory factors in both macrophage and keratinocyte cells presents numerous possibilities for treating various serious inflammatory conditions, including skin diseases. Two studies demonstrated that allergic diseases follow a specific sequence, beginning with atopic dermatitis and food allergies in infancy and progressing gradually to allergic asthma and rhinitis in childhood [29,30]. However, the mechanisms underlying the so-called atopic march remain incompletely understood, given the complexity and heterogeneity of atopic diseases, which result from a combination of genetic, environmental, and epigenetic factors [29]. 

The UPLC-MS/MS analysis revealed that fatty acids and their oxygenated derivatives were the predominant class of lipids in CLE, alongside polar lipids. Among the fatty acids identified, MUFA ω-9 oleic acid (OA) and PUFA ω-3 stearidonic acid (SDA) were the only ones found in their free form. Studies describing the anti-inflammatory activity of dietary OA and SDA were found in the literature. OA demonstrated anti-inflammatory effects on LPS-stimulated THP-1 macrophages by blocking the NF-κB pathway and downstream production of pro-inflammatory cytokines, also promoting the production of the anti-inflammatory cytokine IL-10 [72]. In a study by Sung et al., it emerged that SDA also exerted anti-inflammatory effects via the inactivation of the NF-κB signaling pathway, suppressing iNOS-mediated NO production [73]. 

Oxygenated fatty acid derivatives were found in CLE, which included PUFA oxylipin derivatives and hydroxylated derivatives of saturated fatty acids. Oxylipins are interesting metabolites derived from PUFAs and are nowadays recognized as being involved in the defense mechanisms of macroalgae [74]. Indeed, since an acquired immune system is missing in macroalgae, they heavily rely on their secondary metabolites to mediate interactions with other organisms and the environment, and it was suggested that seaweed oxylipins, like mammals’ leukotrienes and prostaglandins, have a role in their systemic defense mechanisms [42]. As members of *Chlorophyta* are typically rich in C18 PUFA [75], they mainly oxidize C18 substrates [42]. Accordingly, oxylipin derivatives found in CLE were the LA 18:2ω-6 derived hydroxy-linoleic (13-HODE) acid and the ALA C18:3ω-3 derivative 9(10)-EpODE, also known as 9(10)-epoxy-12Z,15Z-octadecadienoic acid. To the best of our knowledge, this is the first evidence of the presence of 9(10)-EpODE in *C. linum*, while a previous report of HODE fatty acid derivatives in this species was found in the literature [76]. Of note, the antinociceptive role of ω-3 epoxy-fatty acids, including 9(10)-EpODE, has been reported in a study on monoepoxides from eicosapentaenoic and docosahexaenoic acids from different sources [77]. As for the second-mentioned oxylipin found in CLE, Kumari et al. reported that in a study comparing the lipidic composition of several different species of macroalgae, *Chlorophyta* contained the highest amounts of hydroxy-oxylipins. Among them, *C. linum* presented a predominance of octadecanoids (C18-oxls) derived from C18 PUFAs (namely, linoleic acid [LA; C18:2ω6] and α-linolenic acid [ALA; C18:3ω3]), including hydroxy fatty acid 13-HODE [76], in accordance with what we found in CLE. This could prove to be very interesting since the esterified form of hydroxylated fatty acids is a recently discovered class of biologically active lipids whose role in inflammation and diabetes in mammals has been indicated to be very attractive [78]. Indeed, an LA ester of 13-HODE found in both mammals and plants has been reported to have anti-inflammatory activity in an LPS-induced cytokine secretion assay [79].

Polar lipid Palmitin, a palmitic acid (16:0) derivative, was also identified in CLE. In general, seaweed-derived fatty acids are mainly found as polar lipids, such as glycolipids (GL), which include monoacyl-, diacyl-, and triacylglycerol (MAG, DAG, and TAG) subclasses, and phospholipids (PL), but also betaine lipids. MAG is formed from TAG and DAG by hydrolysis, which also generates free fatty acids [75]. Although Stabili et al. previously reported the presence of glycerol moieties of monoacyl, diacyl, and triacylglycerol in *C. linum* from Mar Piccolo [39], to our knowledge, this is the first report of the identification of Palmitin in this species. Palmitin has recently been identified in the ethanolic extract of *Spirulina platensis*, a well-recognized superfood with extensively documented beneficial properties, as one of the metabolites responsible for the inhibition of NO production via the downregulation of iNOS, TNF-α, and IL-6 in LPS-induced BV2 microglia [80]. Moreover, it was detected in a methanolic extract of red seaweed *Kappaphycus malesianus*, which prevented the release of cytokines and other pro-inflammatory mediators by inhibiting the NF-κB pathway during microglia-mediated neuroinflammation [81]. 

In silico studies also revealed the potential anti-inflammatory role of palmitic acid in CLE. Despite its known pro-inflammatory nature, the findings of this investigation suggested that it can act as an agonist with biological targets within the receptor complement system considered here. Notably, palmitic acid’s anti-inflammatory properties may result from its interaction with the phosphodiesterase enzyme family [82,83]. Previous research has shown the inhibition of various phosphodiesterase isoforms by free fatty acids [84], indicating that fatty acids could mitigate inflammatory processes mediated by this enzyme family, thus supporting the computational findings. Through docking simulations, palmitic acid demonstrated a high binding free energy score, forming robust polar interactions with the binding residues of known inhibitors of the target, sharing the same binding pocket [85,86].

The second most abundant class of secondary metabolites found in CLE was amino acids. Extensive data report a high content of proteins in *C. linum* [7,87,88]. Of note, the main nutritional value of green seaweeds is in their protein and carbohydrate content, and the composition of essential amino acids (EAA) especially determines the nutritional quality of such proteins [4]. Indeed, green seaweed proteins are known to contain an amount of EAA close to casein and legume proteins, recognized as the principal sources of EAA. One important EAA is Valine, which was among the identified amino acids in CLE as well as in *C. linum* from Mar Piccolo of Taranto, as previously reported by Stabili et al. [39]. Besides their nutritional value, seaweed proteins and peptides have also demonstrated remarkable antioxidant, antimicrobial, antitumoral, anti-hypertensive, anti-diabetic, and anti-inflammatory properties [89,90,91,92,93,94,95]. Among the proteins found in seaweed, lectins are more broadly characterized for their anti-inflammatory activity, but numerous studies have reported the ability of other seaweed-derived proteins and peptides to interfere with inflammatory pathways. These studies, mainly conducted in vitro, included inhibition of NF-κB in macrophagic models, resulting in a decrease in the production of COX-2, iNOS, and TNF-α [96,97,98,99,100].

The other two classes of compounds found in CLE through UPLC-MS/MS analysis were flavonoids and terpenoids. While flavonoids are renowned for their anti-inflammatory properties [101,102,103,104,105,106], terpenoids, particularly carnosic acid, also exhibit anti-inflammatory potential [107,108,109].

Based on in vitro studies and metabolic profiling, we were confident that CLE exerts anti-inflammatory properties. All these findings strongly suggest that CLE may regulate the release of inflammatory markers by blocking the NF-κB inflammatory pathway. However, additional research is required to elucidate specific inhibitory mechanisms and cellular targets of the found active metabolites to further understand how each interacts with the other, acting as parts of a complex anti-inflammatory matrix.

## 4. Materials and Methods

### 4.1. Materials

Dulbecco’s Modified Eagle’s Medium (DMEM), trypsin solution, and all the solvents used for cell culture were purchased from Merck (Darmstadt, Germany). Mouse immortalized fibroblasts (NIH3T3) and RAW 264.7 cells were from the American Type Culture Collection (Manassas, VA, USA). Ames test kit was supplied by Xenometrix (Allschwil, Switzerland).

### 4.2. The Collection of Chaetomorpha linum and the Preparation of Algae Extract (CLE)

The algae *C. linum* (Müller) Kützing was collected from the Orbetello Lagoon (Tuscany, Italy) in May 2021 (Table 3). The samples were harvested and then rinsed with water to remove salt and debris. After being thoroughly cleaned, the material was oven-dried at 55 °C until it reached a constant weight. It was then ground into a fine powder, and the pulverized algae were extracted with an ethanol–water (70:30 *v*/*v*) mixture using a sample/solvent ratio equal to 1:10 (g/mL) at 80 °C for 3 h. The supernatant was then separated from residual biomass, filtered, and subjected to rotary evaporation to remove organic solvent. Finally, the aqueous residue was freeze-dried to produce the dry extract. The extraction was carried out in duplicate. Following extraction, 100 mg of dry extract was dissolved in 1 mL of 100% DMSO to obtain a 100 mg/mL CLE stock solution. This solution was then aliquoted and stored in the refrigerator at −32 °C for subsequent analyses.

### 4.3. NMR Spectroscopy

Samples for NMR spectroscopy were prepared by dissolving 1 mg/mL of CLE dry extract in DMSO-d6 (Cambridge Isotope Lab, Cambridge, MA, USA). Spectra were acquired on a Bruker Avance III 600 spectrometer operating at 14.1 T using a spectral with 8000 Hz and digitized over 32k points by accumulating 32 transients with a recycle delay of 5 s. The residual signal from DMSO was used as a chemical shift reference. Spectra were processed and analyzed using Chenomx 10 (Chenomx Inc., Edmonton, AB, Canada).

### 4.4. UPLC-MS/MS

To investigate the non-volatile profile of CLE, an Ultimate 3000 UPLC system (Thermo Fisher Scientific, Waltham, MA, USA) was used and was controlled using Thermo Xcalibur software (Thermo Fisher Scientific, Waltham, MA, USA). The dry *Chaetomorpha linum* extract was dissolved in the ethanol–water (70:30 *v*/*v*) mixture and injected into the UPLC-Q-Exactive plus system. The samples were separated using a column Acquity UPLC BEH C18 (2.1 mm × 15 cm, 1.7 μm, Waters, Waltham, MA, USA). Mobile phases consisted of solvent A (0.1% formic acid in water) and solvent B (0.1% formic acid in acetonitrile). The gradient started with 2% of B, which was maintained constant for 1 min. Then, the organic phase was increased up to 100% in 50 min. Phase B was maintained at 100% for another 2 min and then returned to the initial condition. The flow rate was maintained at 0.2 mL/min, and the injection volume of the sample was 10 μL. Additionally, the column temperature was kept at 35 °C. A Q-Exactive Plus™ quadrupole Orbitrap mass spectrometer (Thermo Fisher Scientific, Waltham, MA, USA) was used to perform mass spectrometry analyses in the negative and positive ion modes, with a scan mass range set at *m*/*z* 200–2000. HR-MS spectra were recorded in the positive and negative ion modes using the following parameters: spray voltage 3.5 kV (positive) and 3.0 kV (negative), sheath gas 20 (arbitrary units), auxiliary gas 5.0 (arbitrary units), capillary temperature 320 °C, and resolution 35,000. MS/MS spectra were obtained by a Higher Energy Collision Dissociation (HCD) of 30 (arbitrary units). The accuracy error threshold was fixed at 5 ppm. The final annotated metabolome dataset was generated by Compound Discoverer 3.3 (Thermo Fisher Scientific, Waltham, MA, USA). The Compound Discoverer 3.0 software is fully integrated with the ChemSpider database and the mzCloud for automated and expedited data processing. The retention time tolerance (RT) was set to 0.2 min, with mass tolerance equal to 5 ppm, and other parameters were selected as the default values for peak extraction and peak alignment.

### 4.5. GC-FID

The fatty acids profile of CLE was assessed using GC-FID with a Perkin Elmer Clarus 500 gas chromatograph equipped with a flame ionization detector (FID) and a split/splitless inlet. Detector and inlet temperatures were set at 230 °C and 280 °C, respectively. Helium served as the carrier gas at a flow rate of 1 mL/min. The split ratio was 1:10, and the injection volume was 1 µL. A SPTM-2380 fused silica capillary column (60 m × 0.25 mm I.D., Supelco, Bellefonte, PA, USA) was used. The oven temperature was programmed from 70 to 120 °C at 10°/min, increased to 243 °C at 2 °C/min, followed by another increase to 260 °C at a rate of 15 °C/min and held at 260 °C for 20 min. Data acquisition was performed using Perkin Elmer TotalChrom Navigator 6.3.1 software. The CLE was prepared for GC-FID analysis by converting triglycerides to fatty acid methyl esters (FAME) with 14% BF3 methanolic solution from Sigma-Aldrich S.r.l. (Milan, Italy). To the sample (3 mg), 1 mL of BF3 solution was added and kept for 30 min at 95 °C. After cooling, the sample was extracted with 1 mL of n-exane and centrifuged for 5 min (3400 × *g*). The n-exane phase was used for GC analysis after dilution with n-exane (1:10 *v*/*v*). Analyses were conducted in triplicate. Compound identification was performed by comparing chromatographic peaks with those of a 37-component FAME Mix from Sigma-Aldrich S.r.l. (Milan, Italy) analyzed in the same chromatographic conditions.

### 4.6. In Vitro Anti-Inflammatory Activity

#### 4.6.1. Cell Cultures 

RAW 264.7 and HaCaT cells were obtained from ATCC (ATCC, Manassas, VA, USA) and cultured in DMEM containing 10% *v*/*v* FBS, 100 mg/mL penicillin, and 100 mg/mL streptomycin. Cultures were maintained at 37 °C in a humidified atmosphere of 5% CO_2_. Comparative analysis was performed with cell populations at the same generation.

#### 4.6.2. RAW 264.7 and HaCaT Cells Viability

RAW 264.7 cells were seeded at a density of 1 × 10^4^ cells/well in 96-well plates and cultured until sub-confluence (80–85% confluence). Cells were treated with different concentrations (6, 12, 25, 50, and 100 µg/mL) of CLE prepared in DMSO (Sigma-Aldrich) and diluted in medium, and the final DMSO concentration was kept below 0.1% *v*/*v* throughout the experiment. The control was treated with DMSO at a concentration of 0.1% *v*/*v*, corresponding to the highest concentration of the compound. After 24 h of treatment, cells were washed with sterile PBS, and MTT was added to a final concentration of 1 mg/mL. After a 2 h incubation, cells were lysed with 150 µL of DMSO. The absorbance was measured at 550 nm using an EnVision system (PerkinElmer, Waltham, MA, USA), and the percentage of cell viability was calculated relative to the control. The percentage of cell viability was calculated relative to the control. The viability of HaCaT cells was measured using the Cell Counting Kit-8 (CCK-8) (Sigma-Aldrich, Burlington, MA, USA) according to the manufacturer’s instructions. The viability of cells, treated as before, was measured at 450 nm using a microplate reader (CLARIOstar, BMG Labtech, Ortenberg, Germany). The percentage of viable cells was determined relative to the vehicle control.

#### 4.6.3. Cell Stimulation

RAW 264. 7 and HaCaT cells were treated with CLE for 4 h prior to 24 h stimulation with Lipopolysaccharide (LPS) (obtained from Escherichia coli O111:B4, Sigma-Aldrich) or TNFα/IFN-γ (Sigma-Aldrich), respectively. Dexamethasone (DEX) (Sigma-Aldrich), commonly used to treat inflammation, was used as a positive control at a concentration of 5 µg/mL. 

#### 4.6.4. The Quantification of Intracellular ROS Generation

The generation of ROS in RAW 264.7 cells was determined in 96-well plates with 2′,7′-dichlorodihydrofluorescein diacetate (DCFH_2_-DA, Sigma-Aldrich), which was intracellularly deacetylated and oxidized to highly fluorescent 2′,7′-dichlorofluorescein (DCF) [110]. After pre-treatment with different concentrations of CLE, cells were stimulated with LPS (200 ng/mL) for 5 h. DCFH_2_-DA (10 µM) dissolved in HBSS was applied to the cells and incubated at 37 °C. The plate was scanned using an EnVision system (PerkinElmer) with an excitation wavelength of 485 nm and an emission wavelength of 535 nm. Afterward, the number of cells in each well was determined by Crystal Violet assay [111]. The results were normalized to the relative cell count for each well and expressed as the relative ROS production % (RFI) with respect to the LPS group.

#### 4.6.5. The Determination of NO Production

The production of nitric oxide (NO) in the supernatant of RAW 264.7 cells was determined in 6-well plates (1 × 10^6^ cells/well) cultured until sub-confluence (80–85%). After treatment with CLE at different concentrations (25, 50, and 100 μg/mL) for 4 h, the cells were stimulated with LPS (200 ng/mL) for 24 h. Following stimulation, 100 µL of conditioned medium from each well was transferred to a new 96-well plate and mixed with an equal volume of Griess reagent composed of 1% sulfanilamide and 0.1% N-(1-naphthyl) ethylenediamine dihydrochloride in 5% phosphoric acid. After incubation at room temperature for 10 min, the absorbance was measured at 540 nm using an EnVision system (PerkinElmer). Nitrite concentration was assessed by a sodium nitrite standard curve. 

#### 4.6.6. Immunofluorescence Study

RAW 264.7 cells were pre-treated with CLE at 100 μg/mL for 4 h and stimulated with LPS for 1 h. The cells were fixed with 4% paraformaldehyde dissolved in PBS for 15 min and then permeabilized with 0.5% TritonX-100 in PBS for 5 min. After blocking, cells were incubated overnight at 4 °C with anti-NF-κB p65 (clone 1G10.2) mouse monoclonal antibody (Sigma-Aldrich). Cells were then incubated at room temperature for 1 h with Alexa 594-conjugated goat anti-Mouse IgG (Life Technologies, Carlsbad, CA, USA). Finally, the samples were mounted with a fluoroshield mounting medium with DAPI (Abcam, Cambridge, UK). Images were captured by fluorescence microscopy (Zeiss AxioLabA1, Oberkochen, Germany). The quantitative co-localization analysis of NF-κB p65 and DAPI signals was performed using ImageJ and the JACoP plug-in to determine Manders’ coefficient [112], which represents the percentage of NF-κB p65 pixels that overlap DAPI pixels.

#### 4.6.7. Protein Extraction

Whole-cell lysates were obtained with RIPA buffer, added with phosphate and protease inhibitors, and then disrupted by sonication for 15 min in an ice bath. Protein concentration was assessed using the BCA protein assay. Nuclear fractionations were obtained using the NE-PER™ Cytoplasmic and Nuclear Protein Extraction Kit (Thermo Fisher Scientific, Rockford, IL, USA) according to the manufacturer’s protocol.

#### 4.6.8. Western Blotting 

Twenty micrograms of protein was resolved by 8% SDS–PAGE and transferred onto a nitrocellulose membrane. The membrane was blocked in PBS 10% *w*/*v* nonfat dry milk at RT with gentle shaking for 2 h. The membrane was incubated with anti-iNOS (rabbit polyclonal IgG, 1:10,000 Sigma-Aldrich), anti-COX-2 (rabbit polyclonal IgG, 1:2000 Cell Signaling), anti-NF-κB p65 (clone 1G10.2, 1:500) mouse monoclonal antibody (Sigma-Aldrich), and anti-GAPDH HRP-conjugated (1:50,000) primary antibodies, at 4 °C. The blots were washed three times and incubated with anti-rabbit HRP-conjugated secondary antibody (Sigma-Aldrich) 1:80,000 or anti-mouse HRP-conjugated secondary antibody (Sigma-Aldrich) 1:50,000 for 1 h, RT. After washing three times, immunoreactive bands were detected using ECL (LuminataCrescendo, Merck Millipore, Burlington, MA, USA) and images acquired by LAS4000 (GE Healthcare, Chicago, IL, USA). The optical densities of immunoreactive bands were analyzed by ImageQuantTL software (GE Healthcare, Chicago, IL, USA, V 7.0) using GAPDH as a loading normalizing factor.

#### 4.6.9. Enzyme-Linked Immunosorbent (ELISA) Assay 

RAW 264.7 and HaCaT cells (5 × 10^6^ cells/mL) were seeded in 6-well plates and cultured for 24 h. After treatment with CLE at different concentrations (25, 50, and 100 μg/mL) for 4 h, the cells were stimulated with LPS (200 ng/mL) or TNF-α/IFN-γ (10 ng/mL) for 24 h. DEX (5 µg/mL) was used as a positive control. Then, the culture supernatants were collected. The concentration of PGE2 in the supernatants of RAW 264.7 cells was detected using a PGE2 ELISA kit (Cat# E-EL-0034, Elabscience, Houston, TX, USA) according to the manufacturer’s instructions. HaCaT cells’ supernatants were analyzed using ELISA kits for TARC (EHCCL17, Invitrogen, Waltham, MA, USA), RANTES (EHRNTS, Invitrogen), Human IL-8 (BMS204-3, Invitrogen), Human Il-1β (RAB0273, Sigma Aldrich), and MCP-1 (BMS281, Invitrogen) according to the manufacturer’s instructions.

### 4.7. Mutagenicity Assay: Ames Test

The TA100 and TA98 strains of Salmonella typhimurium were utilized for mutagenicity assay in the absence and presence of metabolic activation, i.e., with and without S9 liver fraction. The tester strains used were selected because they are sensitive, detect a large proportion of known bacterial mutagens, and are most commonly used routinely within the pharmaceutical industry [113]. The following specific positive controls were used, respectively, with and without S9 fraction: 2-Nitrofluorene (2-NF) 2 µg/mL + 4-Nitroquinoline N-oxide (4-NQO) 0.1 µg/mL, and 2-aminoanthracene (2-AA) 5 µg/mL. The final concentration of S9 in the culture was 4.5%.

Approximately 107 bacteria were exposed to 6 concentrations (0.025, 0.050, 0.10, 0.50, 1.0, and 10.0 mg/mL) of the CLE extract, as well as to positive and negative controls, for 90 min in a medium containing sufficient histidine to support approximately two cell divisions. After 90 min, the exposure cultures were diluted in a pH indicator medium lacking histidine and aliquoted into 48 wells of a 384-well plate. Within two days, cells that had undergone the reversion to His grew into colonies. Metabolism by the bacterial colonies reduced the pH of the medium, changing the color of that well. This color change can be detected visually. The number of wells containing revertant colonies was counted for each dose and compared to a zero dose control. Each dose was tested in six replicates. The material was regarded mutagenic if the number of histidine revertant colonies was twice or more than the spontaneous revertant colonies.

### 4.8. Statistical Analysis 

Experiments were performed in triplicate. Statistical analyses were performed using GraphPad Prism 9.0 software (GraphPad Software, San Diego, CA, USA). Data were presented as mean ± SD and were compared using one-way ANOVA with appropriate post hoc test. A *p*-value of 0.05 or less was considered significant.

### 4.9. In Silico Studies

#### Structural Optimization and Resources

The anti-inflammatory target complement was retrieved from DrugBank [114] using the “target section” with the keyword “inflammatory”. To select the RAW 264.7 and HaCaT anti-inflammatory target complement, we analyzed the RAW 264.7 transcriptome using the Harmonizome 3.0 database [115] and extracted all targets present in the anti-inflammatory target complement list of DrugBank. Their 3D structures and FASTA sequences were retrieved from the RCSB Protein Data Bank [116] and UniProt database [117], respectively. The 3D structures were obtained by performing multiple sequence alignments with BLASTp v.2.15.0 (National Center For Biotechnology Information, MD, USA) and choosing PDB as the search database; all parameters were used as default [118]. All targets considered in this study are reported in Table S1.

To avoid errors during the docking simulations, potential missing side chains and steric clashes in the 3D structures reported in PDB files were added/resolved with molecular/homology modeling using MODELLER v.9.3 implemented in PyMOD3.0 (PyMOL2.5 plugin, Schrödinger, Inc., New York, NY, USA) [119]. The 3D structures were then analyzed and validated using PROCHECK v.3.5.4 (European Bioinformatics Institute, Cambridge, UK) [120]. Prior to conducting the docking simulations, high-energy intramolecular interactions were minimized using GROMACS 2019.3 (Stockholm University and KTH-Royal Institute of Technology, and KTH-Royal Institute of Technology, Stockholm, Sweden) [121] with the charmm36 force field. CHARMM-GUI v.3.8 (Lehigh University, Bethlehem, Palestine) [122] was used to assign all parameters to the biological targets and ligands.

In detail, prior to conducting further simulations, the starting conformation sequence was aligned against its primary structure, allowing for the addition of potential missing side chains to the structure. Furthermore, loop modeling implemented in MODELLER v.9.3 (Departments of Biopharmaceutical Sciences and Pharmaceutical Chemistry, and California Institute for Quantitative Biomedical Research, Mission Bay Byers Hall, University of California San Francisco, San Francisco, CA, USA) was employed to optimize the best starting orientation of each loop within the structure. Lastly, each structure was analyzed using the PROCHECK tool, where a Ramachandran plot (which analyzes the backbone of ϕ and ψ angles and Chi1–Chi2 plots for side chains) confirmed the validity of the starting conformation. Then, we minimized the energy of each structure by performing energy minimization using GROMACS 2019.3 with the charmm36 force field. This step was taken to prevent the possibility of the structures from sterically hindering potential clashes and/or to optimize the energy values. The resulting structures were then immersed in a cubic box filled with TIP3P water molecules, and the system was neutralized with the addition of counter ions. Simulations were run by applying periodic boundary conditions. Energy minimization was performed with 5000 steps using the steepest descent as the algorithm, which converged to a minimum energy with forces less than 10 kJ/mol/nm.

To enhance the reliability of our simulations, we conducted docking simulations based on in vitro evidence. Therefore, we selectively chose targets for which their experimental 3D structures were in complex with an active compound. In cases where multiple 3D structures of the same target were combined with different ligands in different binding regions, such as allosteric pockets, we created a box capable of enclosing such binding regions. Consequently, a box was created for each target, and we set the grid box at the center of mass of the ligand in the experimental 3D structure of the target, using AutoDock/VinaXB v.1.1.2. (Center for Computational Structural Biology (CCSB), La Jolla, CA, USA) and MGLTOOLS v.1.5.7 (The Scripps Research Institute, La Jolla, CA, USA) [123] scripts. To provide a more consistent result for our docking simulation, we changed the default exhaustiveness from 8 to 32 and only selected binding poses with a root mean square deviation (RMSD) 2 Å lower than that of the best-docked pose. All parameters were used as default.

Three-dimensional structures of compounds were retrieved and downloaded in sdf format from the PubChem database [124]. Then, a virtual screening was carried out using the extracted compounds on the targets. OpenBabel v.3.1.0 (University of Cambridge, Cambridge, UK) [125] was used to convert protein and ligand files and to assign gasteiger partial charges, as proposed in previous works [126,127]. The interaction network was explored with the PLIP tool (Biotechnology Center TU Dresden (BIOTEC), Dresden, Germany) [128].

## 5. Conclusions

We identified *C. linum* as a natural source of various bioactive metabolites, making this invasive species from the Orbetello lagoon an attractive subject for further investigation. The CLE extract not only decreased the production of inflammatory mediators in LPS-stimulated RAW 264.7 macrophage cells, potentially through the inhibition of NF-κB nuclear translocation, but it also exhibited an anti-atopic effect by reducing the production of inflammatory chemokines and cytokines in TNF-α/IFN-γ-induced HaCaT cells. These findings suggested that the ethanolic extract from *C. linum* biomass held significant promise as a therapeutic agent for inflammation. By effectively suppressing key inflammatory factors in both macrophage and keratinocyte cells, this research may offer a basis for the identification and development of novel therapeutic anti-inflammatory candidates with fewer adverse effects.

## Figures and Tables

**Table 1:** Matched metabolites in *Chaetomorpha linum* ethanolic extract.

Chemical Class	Name	Retention Time (min)	Formula	Calculated MW	*m*/*z*	Reference Ion	Mass Error (ppm)
Lipids	Palmitin	41.679	C_19_H_38_O_4_	330.2777	331.2849	[M+H]^+^	1.99
	Oleic acid	50.271	C_18_H_34_O_2_	282.2555	281.2482	[M−H]^−^	−1.54
	Stearidonic acid	24.921	C_18_H_28_O_2_	276.2094	277.2167	[M+H]^+^	1.81
	Hydroxymyristic acid	40.396	C_14_H_28_O_3_	244.2033	243.196	[M−H]^−^	−2.14
	Hydroxylauric acid	35.009	C_12_H_24_O_3_	216.172	215.1647	[M−H]^−^	−2.61
	Dihydroxypalmitic acid	37.957	C_16_H_34_O_4_	290.2461	291.2534	[M+H]^+^	1.38
	9(10)-EpODE	38.428	C_18_H_30_O_3_	294.2204	295.2276	[M+H]^+^	2.9
	Hydroxylinoleic acid	40.866	C_18_H_32_O_3_	296.236	297.2432	[M+H]^+^	2.8
	Lauramide	35.481	C_12_H_25_NO	199.1942	200.2014	[M+H]^+^	2.71
	8-Pentadecenal	41.627	C_15_H_28_O	224.2136	223.2063	[M−H]^−^	−2.03
Amino acids	Valine	2.043	C_5_H_11_NO_2_	117.0793	118.0866	[M+H]^+^	2.5
	Norleucine	1.877	C_6_H_13_NO_2_	131.0948	132.1021	[M+H]^+^	1.18
	Thymine	8.042	C_5_H_6_N_2_O_2_	126.0433	127.0505	[M+H]^+^	2.52
Terpenoids	Carnosic acid	31.801	C_20_H_28_O_4_	332.1984	333.2056	[M+H]^+^	−1.23
	Rosmanol	28.817	C_20_H_26_O_5_	346.1783	347.1856	[M+H]^+^	0.89
	Methyl dehydroabietate	40.729	C_21_H_30_O_2_	314.2241	315.2313	[M+H]^+^	−1.7
	Kaempferol	12.302	C_15_H_10_O_6_	286.0479	285.0407	[M−H]^−^	0.66
Flavonoids	Apigenin	32.899	C_15_H_10_O_5_	270.0532	269.0459	[M−H]^−^	1.42
	3′-O-Methylequol	23.484	C_16_H_16_O_4_	272.1052	271.0979	[M−H]^−^	1.06

Note: MW —molecular weight; EpODE—epoxyoctadecadienoic acid.

**Table 2:** Fatty acid methyl esters identified in CLE.

Fatty Acid	RT (min)	Area % *
Caproic acid C6:0	7.21	1.66 ± 0.19
Caprylic acid C8:0	9.53	0.33 ± 0.06
Lauric acid C12:0	18.68	1.06 ± 0.08
Tridecanoic acid C13:0	20.16	1.38 ± 0.09
Myristic C14:0	23.09	20.46 ± 2.12
Palmitic acid C16:0	29.27	52.18 ± 1.87
Palmitoleic acid C16:1	31.09	1.14 ± 0.15
Stearic acid C18:0	35.51	1.00 ± 0.07
Oleic acid C18:1	37.12	11.70 ± 1.24
Linoleic acid C18:2	39.74	7.35 ± 1.42
Eicosapentanoic acid C20:5	52.62	0.24 ± 0.05
Docosahexaenoic acid C22:6	58.81	2.65 ± 0.18

* % values were expressed in mean ± sd (*n* = 3).

**Table 3:** *Chaetomorpha linum* specimens collected from the Orbetello Lagoon (Tuscany, Italy).

Collection Date	Voucher Number	Type	Species Name	Location	GPS Point
16 May 2021	CL01	Green	*Chaetomorpha linum*	Orbetello	42°26′15.1″ N 11°11′38.7″ E
18 May 2021	CL02	Green	*Chaetomorpha linum*	Orbetello	42°26′15.1″ N 11°11′38.7″ E
25 May 2021	CL03	Green	*Chaetomorpha linum*	Orbetello	42°26′15.1″ N 11°11′38.7″ E

## Data Availability

The original contributions presented in the study are included in the article/Supplementary Material, further inquiries can be directed to the corresponding author.

## Supplementary Materials

The following supporting information can be downloaded at: https://www.mdpi.com/article/10.3390/md22050226/s1, Figure S1: 1D-1H spectrum of CLE dry extract dissolved in DMSO-d6. Peck’s characteristics of saturated and unsaturated fatty acids were labeled with proton originating the resonance underlined. Fatty acid peak intensities over other species were apparent. Table S1: Inflammatory targets.
