# Supplementary figures and images for: Phytochemical Composition, Anti-Inflammatory Property, and Anti-Atopic Effect of Chaetomorpha linum Extract
Source: Mar Drugs. 2024 May 17;22(5):226. doi: 10.3390/md22050226 (PMC11123029; doi:10.3390/md22050226)

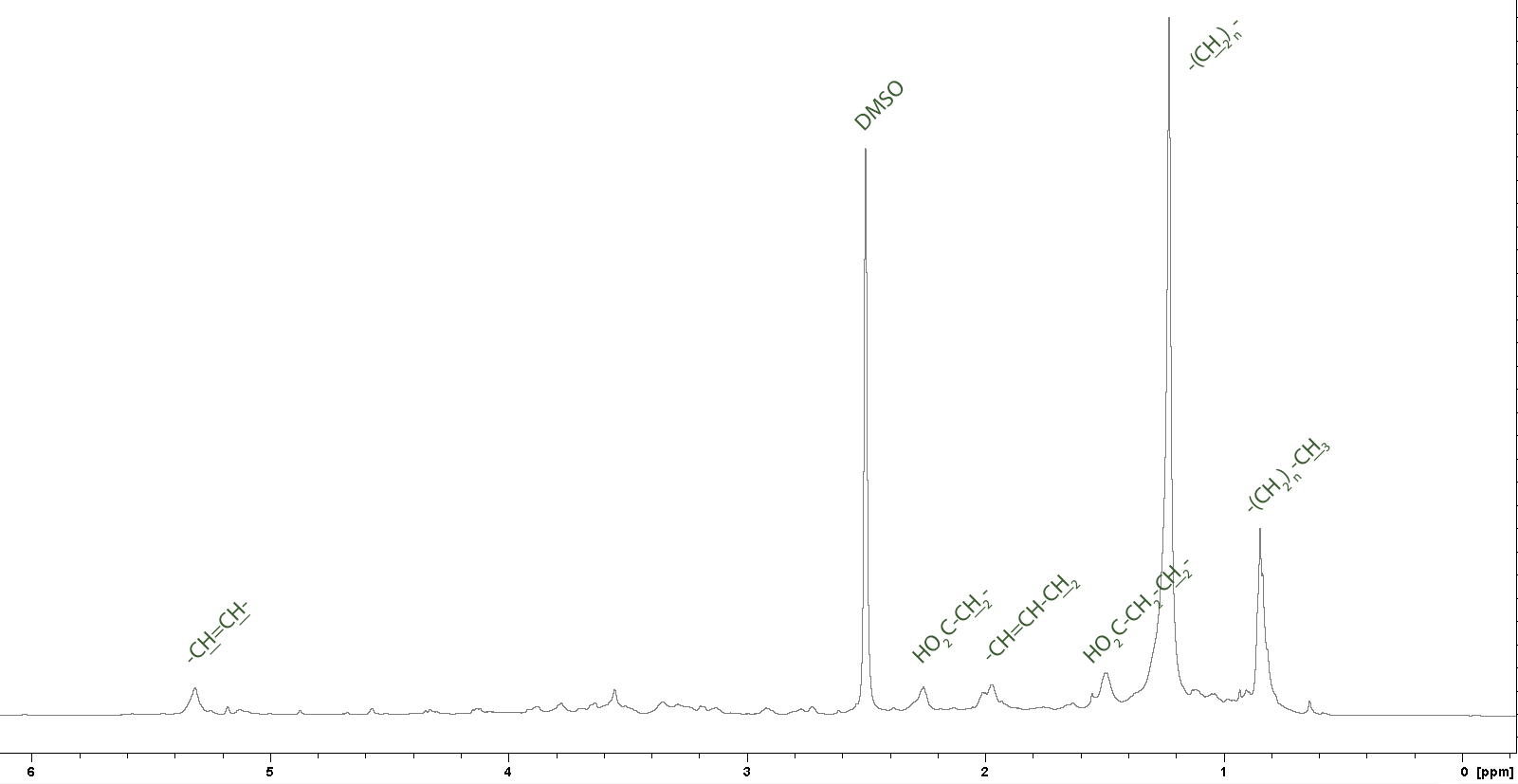

Supplement: Supplementary file 1 [file marinedrugs-22-00226-s001.zip › Supplementary Files/Figure S1.png]
